# Montelukast reduces grey matter abnormalities and functional deficits in a mouse model of inflammation-induced encephalopathy of prematurity

**DOI:** 10.1186/s12974-022-02625-5

**Published:** 2022-10-29

**Authors:** Abi G. Yates, Elena Kislitsyna, Carla Alfonso Martin, Jiaying Zhang, Amy L. Sewell, Ane Goikolea-Vives, Valerie Cai, Lama F. Alkhader, Aleksander Skaland, Basil Hammond, Ralica Dimitrova, Dafnis Batalle, Cathy Fernandes, A. David Edwards, Pierre Gressens, Claire Thornton, Helen B. Stolp

**Affiliations:** 1 Centre for the Developing Brain, School of Biomedical Engineering & Imaging Sciences, King’s College London, London, UK; 2 Icahn School of Medicine at Mount Sinai, New York, NY USA; 3 Comparative Biomedical Sciences, Royal Veterinary College, Royal College Street, London, NW1 0TU UK; 4 Department of Forensic and Neurodevelopmental Science, Institute of Psychiatry, Psychology and Neuroscience, King’s College London, London, UK; 5 SGDP Centre, Institute of Psychiatry, Psychology and Neuroscience, King’s College London, London, UK; 6 MRC Centre for Neurodevelopment Disorders, King’s College London, London, UK; 7 Université Paris Cité, Inserm, NeuroDiderot, Paris, France

**Keywords:** Inflammation, Preterm brain injury, Montelukast, Parvalbumin, Diffusion MRI

## Abstract

Encephalopathy of prematurity (EoP) affects approximately 30% of infants born < 32 weeks gestation and is highly associated with inflammation in the foetus. Here we evaluated the efficacy of montelukast, a cysteinyl leukotriene receptor antagonist widely used to treat asthma in children, to ameliorate peripheral and central inflammation, and subsequent grey matter neuropathology and behaviour deficits in a mouse model of EoP. Male CD-1 mice were treated with intraperitoneal (i.p.) saline or interleukin-1beta (IL-1β, 40 μg/kg, 5 μL/g body weight) from postnatal day (P)1–5 ± concomitant montelukast (1–30 mg/kg). Saline or montelukast treatment was continued for a further 5 days post-injury. Assessment of systemic and central inflammation and short-term neuropathology was performed from 4 h following treatment through to P10. Behavioural testing, MRI and neuropathological assessments were made on a second cohort of animals from P36 to 54. Montelukast was found to attenuate both peripheral and central inflammation, reducing the expression of pro-inflammatory molecules (IL-1β, IL-6, TNF) in the brain. Inflammation induced a reduction in parvalbumin-positive interneuron density in the cortex, which was normalised with high-dose montelukast. The lowest effective dose, 3 mg/kg, was able to improve anxiety and spatial learning deficits in this model of inflammatory injury, and alterations in cortical mean diffusivity were not present in animals that received this dose of montelukast. Repurposed montelukast administered early after preterm birth may, therefore, improve grey matter development and outcome in EoP.

**Supplementary Information:**

The online version contains supplementary material available at 10.1186/s12974-022-02625-5.

## Introduction

Preterm birth occurs in 11% of live births worldwide [1, 2], with 30% of the surviving population suffering from life-long neurological deficits [3–5]. The varied neuropathology, collectively termed encephalopathy of prematurity (EoP), includes grey and white matter injury and increases the incidence of a range of cognitive and motor deficits in later life [6], for which limited treatments are currently available.

Whilst white matter (WM) injury has been the historical focus of preterm brain injury studies, there is increasing evidence of the importance for grey matter (GM) injury in this condition [7]. Neuropathological studies in clinical cohorts, and animal models of preterm injury, have identified decreased neuronal number [8, 9]; dendritic arborisation [10] neurogenesis [11], and developmental alterations in interneuron populations [12, 13]. Clinical magnetic resonance image (MRI) studies have also identified atypical GM development, including smaller cortical volume in EoP [14–16]. Reduced cortical growth from 23 to 45 postmenstrual age (PMA), as assessed by MRI, predicted cognitive deficits at 2 and 6 years of age in preterm-born infants [17]. Diffusion MRI (dMRI) studies have also shown alterations in cortical microstructure assessed with diffusion tensor imaging (DTI) metrics [18, 19], suggesting a potential reorganisation of the developing neurons associated with preterm birth [20].

The underlying causes of EoP are multifactorial and most likely interlinked, with intrauterine growth restriction [21–23], inflammation [24, 25], and chronic hypoxia [26–28] recapitulating aspects of the neuropathology in animal models, and associated with clinical EoP. Microglial activation is well-established in the preterm brain with EoP [29, 30], and long-term epidemiological studies have shown that prolonged systemic elevation of pro-inflammatory cytokines correlates with altered brain development and poor cognitive outcome [24, 31]. Mediators of inflammation regulate many normal processes of brain development (reviewed by [32]) and experimentally induced maternal or systemic inflammation (produced with a variety of inflammatory mediators, including IL-1β) can recapitulate the above mentioned pathological processes of EoP; i.e. activate microglia, reduce neurogenesis, delay myelination, alter neuronal development and cause memory impairment [8, 13, 33–36]. However, to date, therapeutic targeting of either inflammatory or other mechanisms of injury has not been translated to the clinic [37, 38].

The cysteinyl leukotrienes (CysLTs) are a family of highly potent pro-inflammatory mediators, derived from arachidonic acid via the 5-lipoxygenase pathway. They have been associated with a range of neuroinflammatory diseases including cerebral ischaemia [39, 40], Alzheimer’s disease [41], multiple sclerosis [42] and traumatic brain injury [43]. Leukotrienes act through the CysLT receptors (CysLTR), G protein-coupled receptors which are widely expressed throughout the body, particularly on immune cells, and in immune and contractile tissue [44]. In the central nervous system (CNS), the CysLTR1 antagonist, montelukast, has been found to increase neurogenesis [45], reduce microglial activation [46] and blood–brain barrier permeability [47], and support oligodendrocyte development [48], in addition to its immediate anti-inflammatory properties [49]. This data implies that montelukast treatment could ameliorate key pathologies identified in the developing brain subsequent of systemic inflammation [33, 50–52]. Moreover, it has been shown to reduce infarct volume, demyelination, and seizure frequency in adult rodent models of neurological injury [47, 53–56]. Importantly, CysLTs and their primary receptors are not constitutively expressed in the brain [55, 57], and therefore are unlikely to contribute to the neuropoietic cytokine signalling that regulates normal brain development, but their expression is upregulated following injury [53, 58, 59]. As such, we have identified montelukast as a potential therapy for preterm brain injury.

Montelukast is an attractive candidate for repurposing; it is currently utilised as an asthma medication; it has a good safety profile and is approved for administration to infants from 6 months of age. In addition, studies have shown that it is safe and that there are predictable changes in its pharmacokinetics in 0- to 6-month-old infants [60, 61]. Here, we show that concomitant treatment with montelukast reduces central and peripheral inflammation, long-term behaviour deficits and aspects of the underlying GM neuropathology related to EoP.

## Methods

### Animals and drug administration

A model of preterm brain injury was used in this study, whereby exposure to systemic inflammation from postnatal day 1 (P1) to P5 induces diffuse WM injury [33, 34, 52] and selective GM injury [13]. The primary objective of this work was to determine if montelukast was able to ameliorate the inflammatory response, GM injury and behavioural deficits previously reported in this model. All animal experiments were approved by the Animal Welfare and Ethical Review Body (King’s College London) and were carried out in accordance with the regulation and guidance issued by the Animals (Scientific Procedures) Act (1986), observed by Home Office personal and project licences. Data from in vivo experiments have been reported in accordance with the ARRIVE guidelines.

CD-1 time-mated females were purchased from Charles River and were singly housed in individually ventilated SPF cages under standard diurnal lighting conditions (12 h) with ad libitum access to food and water. Consistent with the previous studies using this model, only male pups were used due to a sex bias in this model whereby females do not display myelin deficits [34]. Therefore, female mice were culled following birth, and male mice were randomly and equally distributed into new litters. Litters were randomly divided into 4 treatment groups: (i) saline (SAL), (ii) recombinant mouse IL-1β alone (40 μg/kg, 5 μL/g body weight; R&D Systems, IL1), (iii) IL-1β + montelukast (1, 3, 10, or 30 mg/kg; Sigma Aldrich, IL + MO) or (iv) montelukast alone (MO). Treatments were administered via intraperitoneal (i.p.) injection twice daily from P1–4, and once on P5, during a period of brain development in the mouse which corresponds to weeks 23–32 of gestation in the human [62–64]. Saline or montelukast continued to be administered daily for a further 5 days (i.e. a total of 10 day drug or saline treatment). While each animal was treated individually (representing a distinct biological replicate), all animals in a litter had the same treatment. To avoid bias from litter effects, e.g. maternal care, pups in litters were pseudo-randomly allocated into assessment groups ensuring that groups comprised samples from two or more litters. Tissue was collected for RNA or protein extraction (*n* = 3–9 per group, specific numbers for each experiment detailed in results and figure legends), or histological analysis (*n* = 3–6) at 0.5, 1, 4, 8 and 24 h, as well as at P2, P5 and P10. Twelve animals per group (2/litter) were used for behaviour testing (P36–54), after which tissue was collected at P54 for MRI and histological analysis. Animals from these litters not used for behavioural studies (2–4 per litter) were used at P40 for histological analysis of brain tissue. Animal numbers were based on power calculations (alpha < 0.05, power > 0.8) using previous data from this model [13, 33].

### Tissue collection

Prior to tissue collection animals were terminally anaesthetised with pentobarbital (i.p., 0.1 mL/kg). Blood samples were collected by cardiac puncture using a 25 g needle, for the assessment of plasma drug and cytokine levels. Following collection, blood samples were centrifuged for 5 min at 4000 rpm and the plasma removed. Heavily haemolysed plasma samples were discarded (*n* = 1), the remaining were frozen until use. For the assessment of systemic inflammation, fresh liver was collected and immersed in TRIzol reagent (ThermoFisher) for RNA extraction. Brains were then dissected for mass spectroscopy (fresh frozen), RNA extraction (TRIzol), protein extraction (protein extraction buffer) or for histology following paraformaldehyde perfusion (post-fixation in Bouin’s solution, Sigma Aldrich). Fixed samples were processed to paraffin wax prior to coronal sectioning at 5 μm thickness, using the Leica RM2245 Microtome (Leica Biosystems, Germany).

### Quantitative real time polymerase chain reaction (qRT-PCR)

RNA was extracted from liver and brain samples using RNeasy Mini Kit (Qiagen^©^) as per manufacturer’s instructions. qRT-PCR was completed with 200 ng of RNA, using TaqMan RNA-to-C_T_ *1-Step* Kit (Applied Biosystems), with a StepOnePlus Real-Time PCR system (Applied Biosystems). Relative quantification of expression was determined by the 2^−ΔΔct^ method, normalised to the expression of the housekeeping gene GAPDH, as previously described [65, 66].

### Mass spectrometry

Montelukast quantification in plasma and brain was performed by the Mass Spectroscopy Service at King’s College London. To determine plasma and brain concentration over time, plasma and brain samples of known volume or weight were collected as described above from P5 pups injected with a single 30 mg/kg dose of montelukast. Samples were collected 0.5, 1, 4, 8 and 24 h post injection (*n* = 8–12 per time point). Initial experiments were performed to optimise the mass spectroscopy parameters (based on [67]) to detect montelukast and separate this detection from the degradation product cis-montelukast (using montelukast D-6 as an internal standard). Linearity of concentration in plasma, in the range of 0.5–10 ng/mL was also confirmed. Analysis was performed on a dedicated ACQUITY UPLC BEH C18 2.1 × 50 mm, 1.7 µm particle size column.

### ELISA quantification of CysLT

Protein concentration in the brain was quantified with BCA assay (ThermoFisher). The concentration of cysteinyl leukotrienes (LTC_4_, LTD_4_ and LTE_4_, collectively) was determined for each sample using a competitive ELISA assay, in duplicate and at two dilutions, following manufacturer’s instructions (Cayman Chemicals).

### Immunohistochemistry

Immunostaining was performed using an avidin–biotin–peroxidase (ABP) method, with 3,3′-diaminobenzidine (DAB) for visualisation. Sections were incubated overnight (4 °C) in one of the following primary antibodies, diluted in 1% NHS block: Goat anti-serum albumin (1:5000, Abcam), rabbit anti-parvalbumin (PV, 1:200, Abcam), mouse anti-MBP (1:200, Millipore), mouse-anti-CNPase (1:200, Neomarkers), mouse anti-ICAM-1 (1:200, R&D Systems), goat anti-GFAP (1:300, Abcam) and *Lycopersicon esculentum* (tomato) lectin (1:200, Vector, UK). Biotinylated secondary antibodies (1:200, Vector, diluted in 1% NHS block, 2 h, RT) were used, followed by HRP (ABC Elite, Vector, UK) and DAB prior to imaging and analysis.

For assessment of general tissue structure, sections underwent staining with haematoxylin and eosin (H&E).

### Microscopy and histological image analysis

Micrographs of immunohistochemically stained tissue sections were acquired using a light microscope (Leica DM6000B, Leica Microsystems Ltd.) with bright and fluorescent light capacity, CCD colour video camera (Leica CTR6000, Leica, UK), and equipped with a motorised stage (MicroBrightfield Inc.).

All histological image analysis was performed blinded to treatment group using *Stereo Investigator software* (v8.27 MicroBrightfield Inc., USA) or *ImageJ* (NIH), following semi-automated or manual tools as previously described [13, 27, 33, 51, 68, 69]. Data were averaged from 3 to 6 images per brain (damaged tissue was excluded as was tissue where the staining had excessively high background), and from 3 to 6 brains per treatment and age.

For parvalbumin-positive interneurons, cell counts were performed manually throughout the hippocampus or from a region of interest (ROI) from the sensory (barrel) cortex [13, 33, 51], and data were presented as cells/mm^2^. At P10, arborisation was assessed using an ImageJ classifier to detect parvalbumin-positive arbours within the region of parvalbumin-positive cell. Arbour density was determined relative to total cell number. At P40, when arbours were more clearly delineated, Image J was used to specifically identify arbours attached to immune-reactive cell bodies, and the number and area of identified arbours was assessed. Parvalbumin-positive puncta, indicating synapses from parvalbumin neurons [45], that contacted layer 5 neuronal soma were also measured, as a total and an average number of contacts per cell.

### Magnetic resonance imaging

Ex vivo MRI was performed after PFA perfusion-fixation of tissue, followed by immersion fixation at 4 °C and PBS washing [70]. Diffusion-weighted images were acquired with single-echo EPI sequences with the following parameters: 6, 40, and 60 directions at *b*-values of 1000, 4000 and 8000 s/mm^2^, respectively; an isotropic voxel size of 100 µm with a field of view of 12.8 × 12.8 mm^2^, echo time (TE) = 40.8 ms, and repetition time (TR) = 1000 ms; 13 reference images with *b* = 0 s/mm^2^ were acquired (1, 6, and 6 for each shell, respectively). Total acquisition time was 88 h per subject. Of the 35 scans acquired, five were excluded following visual quality check (2 SAL, 1 IL + MO and 2 MO), due to artefacts produced from air bubbles and/or excessive vibration during the scan. Pre-processing was performed to correct eddy-current, and B1 bias using FSL [71] and MRtrix3 [72]. Diffusion tensor imaging (DTI) was fitted to the *b* = 4000 s/mm^2^ shell data using FSL [73] and FA and MD maps were generated. NODDI metrics were calculated using data from all shells and using the default settings of the NODDI toolbox (http://mig.cs.ucl.ac.uk/index.php?n=Tutorial.NODDImatlab; [74]). A population-based template was generated using multivariate template construction available in ANTS [75], and ROIs were delineated manually in template space and projected back to each subject’s native space. Cortical ROIs were placed in the upper and lower cortex of the sensory cortex, hippocampal ROIs delineated the pyramidal layer (CA_py_), stratum radiatum (CA_sr_) and stratum lacunosum moleculare (CA_slm_) of the CA1 region as well as the dentate gyrus (DG), and white matter ROIs were placed in the corpus callosum (CC), left and right external capsules (EC) and the anterior commissure (AC). Median DTI and NODDI metrics for each ROI were calculated for each subject in native space, and mean values compared between treatment groups.

### Behaviour

A series of behavioural tests were performed to determine whether montelukast was able to alleviate the behaviour deficits observed with this model of preterm brain injury. Three cohorts of animals were tested, with 4 animals per treatment group (2 per litter) in each cohort. Behavioural testing was spread over a 2-week period, with no more than one behavioural test completed each day, after which the animal was returned to their home cage. In all cases the experimenter was blinded to treatment and animals were tested in a pseudo-randomised order, counterbalanced between each behavioural test, and steps taken to ensure testing was performed in a clean, olfactory neutral environment. Data were recorded and analysed with EthoVision tracking software (Noldus) [76].

General locomotor activity and baseline anxiety were tested with the open field paradigm, which measures the conflict between a rodent’s exploratory behaviour and aversion to open, exposed areas [77]. Time spent in the inner area and the distance moved and velocity in the outer region of the arena were determined.

A light–dark box test was used to measure anxiety, based on the conflict between rodents’ exploratory behaviour and aversion to open and brightly lit areas [78, 79]. For testing, each animal was placed into the dark chamber and allowed to explore the apparatus for 5 min. Frequency to enter the light zone, transitions between zones and duration in the light zone were determined.

The Morris water maze (MWM) was performed to assess spatial learning [80, 81]. Animals performed 4 trials per day, with each trial initiating in each of the 4 quadrants in a pre-determined sequence. The path length to the platform, the latency to reach the platform and swimming velocity were determined. Data are presented as performance per day of trial, as well as change across testing period (the difference between performance in the 1st and 6th session).

### Statistical analysis

All statistical analyses were completed with GraphPad Prism 7 software using one- or two-way analysis of variance (ANOVA), as appropriate. Post hoc testing was performed either to compare every mean with every other mean (Tukey’s test), selected pairs of means (Sidak’s test) or every mean to a control mean (Dunnett’s tests) depending on experimental design. Results were considered significant at *p* < 0.05 and reported as statistically significant based on injury or treatment effect, as well as after multiple comparison correction for specific comparisons. Quantitative data are expressed as mean ± standard error of the mean (SEM).

## Results

### Montelukast ameliorates peripheral and central inflammation

**Fig. 1:**
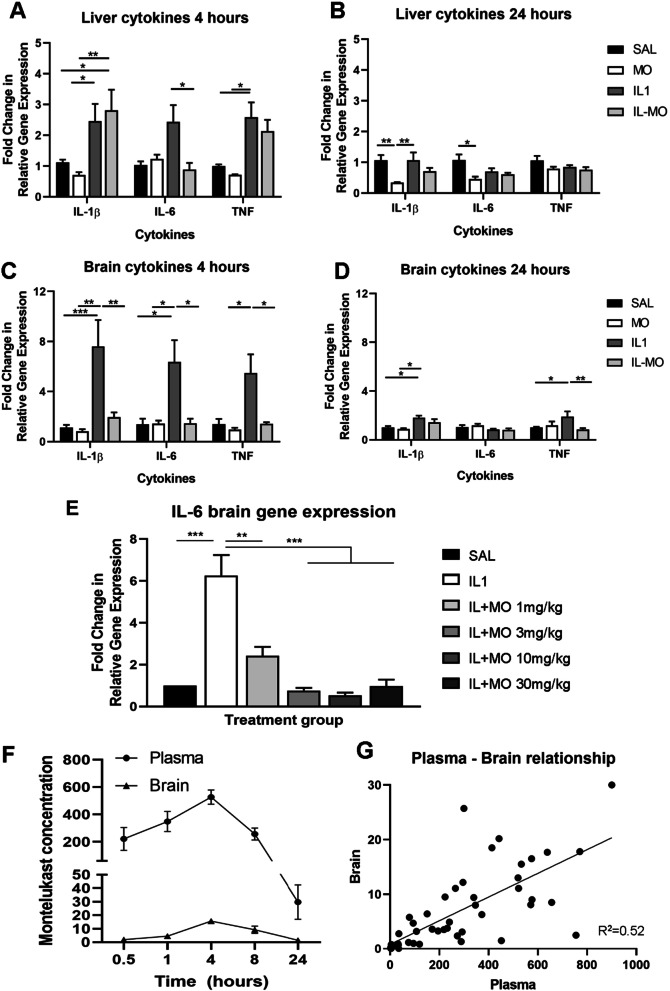
Montelukast reduces peripheral and central inflammation in a mouse model of IL-1β-induced preterm brain injury. Systemic and central inflammation was assessed based on expression level of pro-inflammatory cytokines, IL-1β, IL-6 and TNF, in the liver and brain, respectively, measured by qPCR. The IL-1β treatment group (IL1, *n* = 7) showed increased cytokine expression at 4 h in the liver and the brain, compared to the saline (SAL, *n* = 6) and montelukast alone (MO, *n* = 4) treatment groups (**A**, **C**). These increases consistently returned to control levels following montelukast treatment, both in the brain, and for IL-6 in the liver (IL + MO group, *n* = 6). The cytokine response was substantially reduced by 24 h following the induction of the injury (**B**, **D**), though montelukast treatment still showed some amelioration (SAL *n* = 6, MO *n* = 6, IL1 *n* = 9, IL + MO *n* = 8). Brain IL-6 expression was measured 4 h after induction of systemic inflammation, and concomitant treatment with montelukast at 1, 3, 10 and 30 mg/kg (**E**). The IL-1β-induced increase in IL-6 was significantly reduced by all doses of montelukast in a dose-dependent manner (*p* = 0.0003 for IL + MO 1 mg/kg, < 0.0001 for all other doses, 1-way ANOVA; *n* = 3/group). Mass spectrometry analysis (0.5, 1 and 8 h: *n* = 8; 4 and 24 h: *n* = 12) showed a peak montelukast concentration in the plasma and brain at 4 h post-administration (**F**), with the brain concentration proportional to plasma concentration (*R*^2^ = 0.52, *p* > 0.0001; **G**). Data presented as mean ± SEM, *p < 0.05, **p < 0.01, ***p < 0.001. SAL: saline; IL1: IL-1β; IL + MO: IL-1β + montelukast; MO: montelukast; IL: interleukin; TNF: tumour necrosis factor

In this inflammatory model of preterm brain injury, IL-1β was injected intraperitoneally in male CD-1 mice to provoke a systemic inflammatory response. This in turn induces central inflammation, and the associated structural deficits in the brain [13, 33]. To determine the capacity of montelukast to ameliorate the inflammatory response induced by IL-1β, treatment was administered concomitantly to the IL-1β injection i.p. (simulating treatment from birth in preterm infants) at the high dose of 30 mg/kg. Gene expression in the liver was assessed as a proxy for systemic inflammation [82, 83]. At 4 h after injection of IL-1β, hepatic expression of IL-1β, IL-6 and TNF were increased by an average of 2.5-fold compared with saline-treated animals (Fig. 1A; treatment effect *p* < 0.001, *p* = 0.034 after post hoc test for TNF: SAL vs IL1). At this time point, the increased expression of IL-1β and TNF persisted in animals receiving both montelukast and IL-1β (2.8 ± 0.67-fold and 2.1 ± 0.37-fold, respectively, *p* = 0.022 for IL-1β: SAL vs IL + MO). However, the expression of IL-6 was significantly ameliorated by montelukast (0.88 ± 0.22, *p* = 0.029 IL + MO vs IL1), with expression levels comparable to saline and montelukast controls. By 24 h, the hepatic inflammatory response was substantially reduced, with no significant increase in inflammatory gene expression found in the IL-1β treated animals (Fig. 1B), though a significant reduction in systemic cytokine expression was found in the montelukast group compared to saline control (IL-1β: 0.33 ± 0.02, *p* = 0.004; IL-6: 0.46 ± 0.07, *p* = 0.022).

The central response to the i.p. IL-1β regime was more consistent than the systemic response, and clearly showed the capacity of montelukast to reduce brain cytokine expression. The relative expression of IL-1β (7.60 ± 2.12), IL-6 (6.36 ± 1.74), and TNF (5.47 ± 1.50) were significantly elevated at 4 h compared with saline-treated controls; this elevation was completely ameliorated by high-dose montelukast (to an average value of 1.5-fold; treatment effect *p* < 0.001; SAL vs IL1 *p* < 0.001, *p* = 0.016 and *p* = 0.050 for IL-1β, IL-6 and TNF, respectively; and IL1 vs IL + MO *p* = 0.002, *p* = 0.018 and *p* = 0.042 after post hoc test; Fig. 1C). At 24 h, expression of IL-1β and TNF in the brain remained significantly greater than saline controls (IL-1β: 1.02 ± 0.10 SAL vs 1.83 ± 0.15 IL1, *p* = 0.047; TNF: 1.01 ± 0.06 SAL vs 1.91 ± 0.42 IL1, *p* = 0.020). Montelukast significantly decreased expression of TNF (IL + MO 0.85 ± 0.12, *p* = 0.002, Fig. 1D).

In order to determine how much the central inflammatory response and effect of montelukast was driven by brain concentrations of CysLTs, analysis was performed of both the concentration of CysLTs in the brain and the CysLTR1 (Additional file 1: Fig. S1). qRT-PCR showed a significant increase in the expression of the CysLTR1 24 h following IL-1β treatment, in both the IL and IL + MO groups compared to SAL (Additional file 1: Fig. S1A). At the same time, and also at the end of the IL-1β treatment period (P5), ELISA analysis detected an increase in CysLTs in the brain tissue in the IL-1β group, though this was only statistically significant at P5 (*p* < 0.05). At both ages, the IL–MO group was not significantly different from either the SAL or IL1 group (Additional file 1: Fig. S1B, C).

Next, we sought to identify the lowest effective dose in this inflammatory model of preterm brain injury. As a marker of central inflammation, relative gene expression of IL-6 in the brain was measured 4 h post-injury (Fig. 1E). Results demonstrated a significant increase in IL-6 in the brains of IL-1β-treated mice, showing a sixfold increase in expression in comparison to saline-treated group (*p* < 0.001).

Montelukast significantly reduced IL-6 expression in a dose-dependent manner; administration of the 1 mg/kg dose reduced expression significantly compared to the injury group (*p* = 0.007), and was not significantly different from the control, despite remaining 2.5-fold higher than the saline only group (*p* = 0.295, Fig. 1E). Administration of montelukast at 3, 10 and 30 mg/kg each prevented the increase in expression, with IL-6 expression levels comparable to the saline only group (*p* = 0.99) and significantly different from the injury group (*p* < 0.001). As such, 3 mg/kg was determined as the lowest dose completely effective at reducing central inflammation.

To determine whether montelukast could exert its effect directly in the brain, mass spectrometry of plasma and brain tissue was completed following i.p. administration in postnatal mice. Data showed that there was a peak in plasma concentration at approximately 4 h (Fig. 1F). A peak in brain concentration occurred at the same time, proportional to the plasma concentration (*R*^2^ = 0.52, *p* < 0.001; Fig. 1G). Detection of montelukast in the brain was independent of BBB breakdown (Additional file 1: Fig. S2A), although vascular inflammation was evident (Additional file 1: Fig. S2B, C), suggesting a small proportion of montelukast moves passively into the brain following systemic administration.

### Inflammation-induced deficits in the grey matter are ameliorated by montelukast

**Fig. 2:**
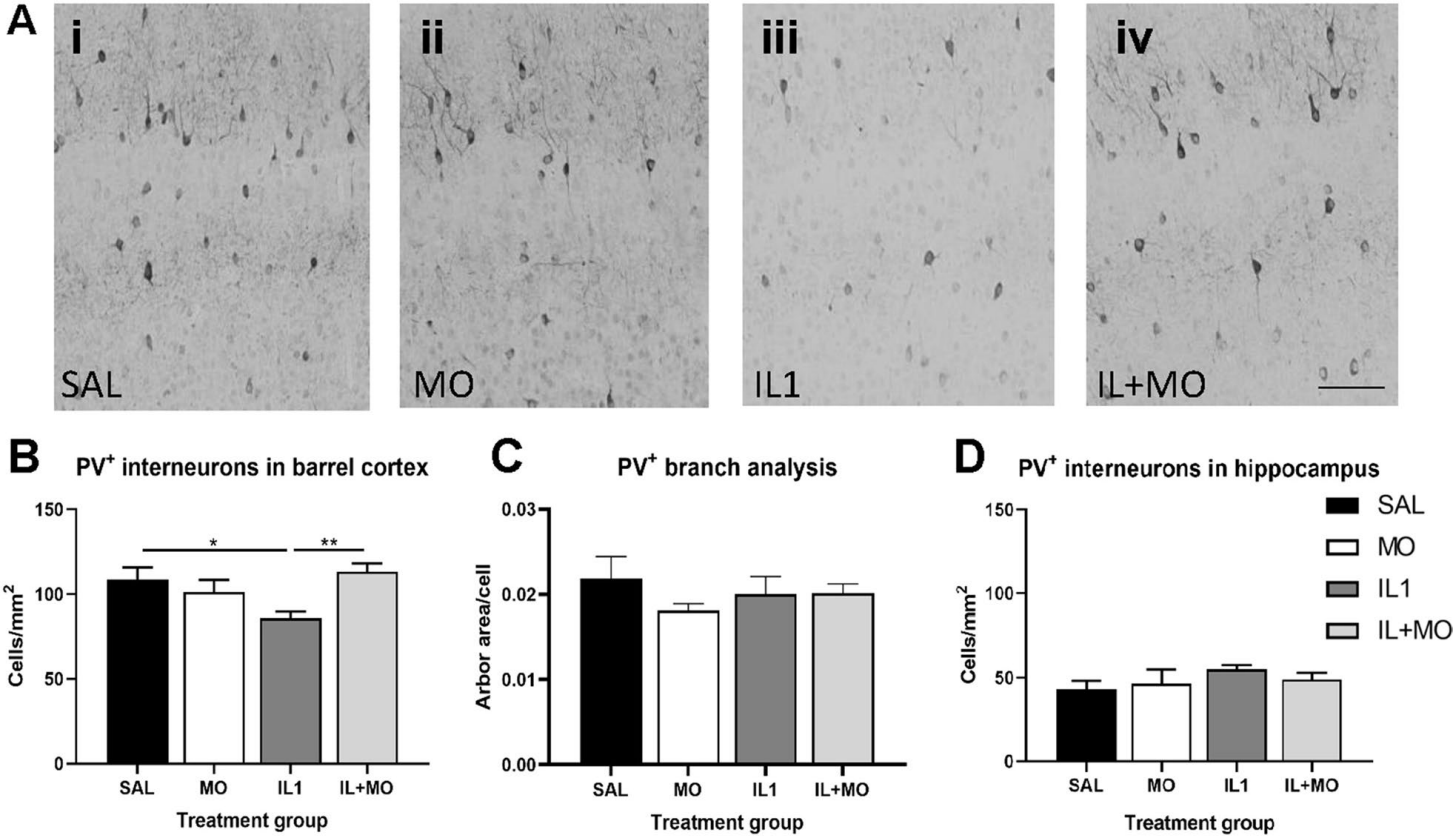
Inflammation-induced parvalbumin deficit at P10 was ameliorated by montelukast. Immunohistochemistry for parvalbumin was used to assess the capacity of montelukast to ameliorate histopathological changes associated with the GM (**A**). Inflammation induced a decrease in parvalbumin interneurons in the barrel cortex, which was restored by 30 mg/kg montelukast treatment, with cell densities returning to control levels (**B**). This reduction in cell number was not accompanied by a change in branching of the remaining cells (**C**). No change with inflammation or montelukast treatment was seen for hippocampal parvalbumin interneurons (**D**). Data presented as mean ± SEM, *p < 0.05, **p < 0.01. Scale bars: **A** = 500 µm; SAL: saline (*n* = 6); IL1: IL-1β (*n* = 8); IL + MO: IL-1β + montelukast (*n* = 6); MO: montelukast (*n* = 5)

Abnormalities in the GM have been reported in both animal models [10, 84] and human infants [85, 86] with preterm brain injury, including an interneuronopathy affecting parvalbumin neurons recently identified in this animal model [13]. As such, we investigated the effect of inflammation and high-dose (30 mg/kg) montelukast treatment on parvalbumin-positive (PV^+^) interneurons at P10. The high dose of montelukast was used to determine the maximum capacity for montelukast to protect from short-term cellular injury. The density of PV^+^ interneurons were significantly decreased in the barrel cortex in IL-1β-treated animals (85 ± 4 cells/mm^2^) compared to saline controls (109 ± 7 cells/mm^2^, *p* < 0.027, Fig. 2Ai–iv, B). This deficit was ameliorated by treatment with montelukast, with cell density returning to control levels (113 ± 5 cells/mm^2^, *p* = 0.006). While there was a change in cell number, no alterations in the arborisation of the existing PV^+^ interneurons were detected (Fig. 2C). In contrast to the findings in the cortex, no significant alteration in the number of PV^+^ interneurons was found in the hippocampus (Fig. 2D).

### Long-term neurostructural changes are ameliorated by montelukast in the cortex

**Fig. 3:**
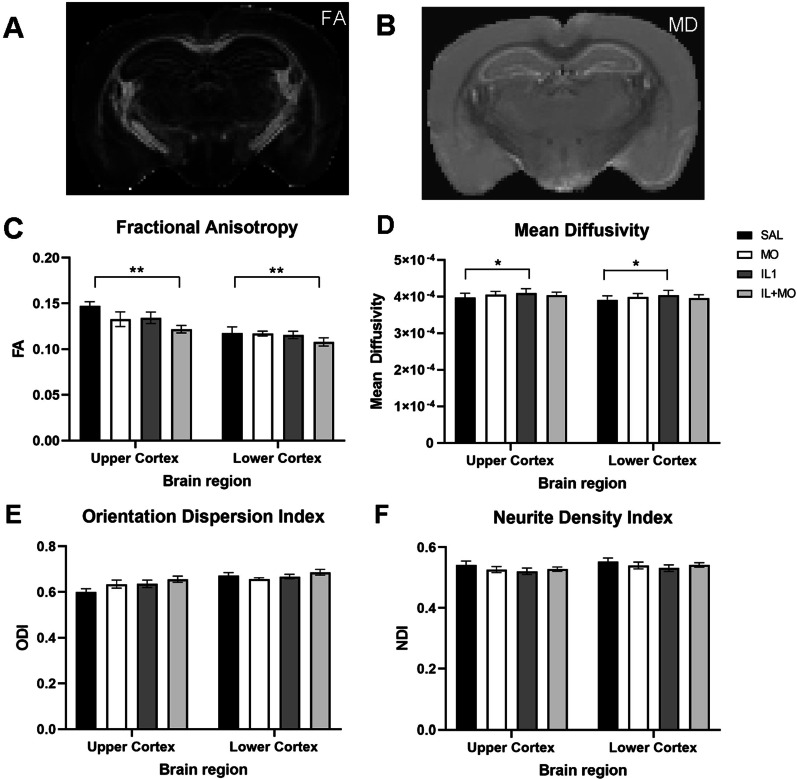
Cortical microstructural changes can be detected long-term using diffusion MRI. High-resolution multi-shell ex vivo diffusion MRI was performed to determine if early brain injury persisted into adulthood. The diffusion tensor (DTI) model was applied to the b-4000 shell to derive the fractional anisotropy (FA; **A**, **C**), and mean diffusivity (MD; **B**, **D**). The NODDI model was applied using data from all shells and orientation dispersion index (ODI, **E**) and neurite density index (NDI, **F**) were quantified. A statistically significant decrease was determined in FA between control and IL–MO groups for the upper and lower cortex. Whereas, for MD a statistically significant increase was identified between the saline and IL-1 treatment groups in these regions. Data presented as mean ± SEM, *p < 0.05, **p < 0.01. SAL: saline (*n* = 7); IL1: IL-1β (*n* = 9); IL + MO: IL-1β + montelukast (*n* = 8); MO: montelukast (*n* = 6)

We performed a diffusion MRI and neuropathological assessment of the cortical GM to determine any long-term structural effects of postnatal inflammation and low-dose (3 mg/kg) montelukast as a potential therapy. Assessment of FA (Fig. 3A, C) showed a significant difference between brain region (upper and lower cortex, *p* < 0.0001), in addition to a significant difference between treatment groups (*p* = 0.018). Following post hoc analysis assessing effect between treatment groups, a significant decrease in FA was found between the saline group and the group treated with IL-1β and montelukast (SAL: 0.147 ± 0.011, IL + MO: 0.121 ± 0.011, *p* = 0.01).

MD showed statistically significant differences between brain regions (*p* = 0.028) and treatment group (*p* = 0.025, Fig. 3B, D). The average MD in the cortex of saline-treated animals was 3.9 × 10^–4^ ± 0.01 × 10^–4^ mm^2^/s, which increased to 4.1 × 10^–4^ ± 0.01 × 10^–4^ mm^2^/s in the IL-1β treated animals (*p* = 0.014) and was the only difference statistically significant after post hoc testing. MD in IL + MO was not significantly different from SAL or IL animals. Orientation dispersion index (ODI) and neurite density index (NDI) showed median values per group inversely proportional to those calculated for FA and MD, respectively (Fig. 3E, F), but were not significantly different following post hoc statistical analysis.

**Fig. 4:**
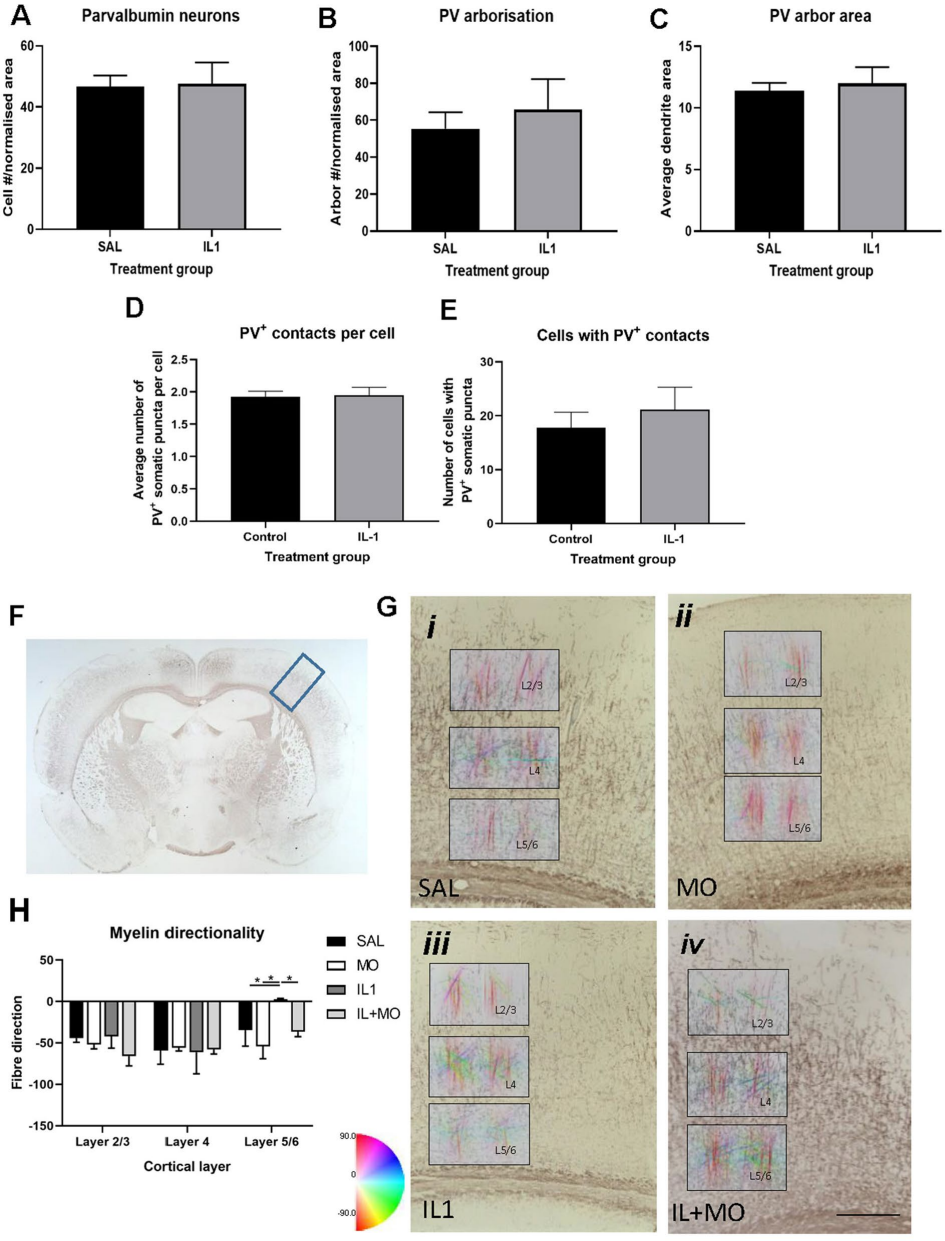
Limited microstructural changes were observed in the cortex long-term. At P40, no change in the number of parvalbumin cells (**A**), their arborisation (**B**, **C**) or the number of detected synapses (**D**, **E**) were found. Directionality analysis of axonal fibres protruding into the cortex, identified from myelin basic protein immunohistochemistry (**F**, **G**), did show a significant difference in layer 5 of the cortex, but not upper layers (**H**, 2-way ANOVA). Data presented as mean ± SEM, *p < 0.05. Scale bars **G** = 100 µm. SAL: saline (*n* = 3); IL1: IL-1β (*n* = 3); IL + MO: IL-1β + montelukast (*n* = 4); MO: montelukast (*n* = 3)

Histological analysis of the cortex was subsequently performed to determine whether altered interneuron morphology or cortical myelination might explain the MRI findings. Previous work from our lab has shown that PV^+^ neuronal number recovers by P40 [13], which was confirmed in this dataset (Fig. 4A). In order to determine if there were more subtle differences in the microstructure of the cortex, an analysis of the arborisation of PV^+^ neurons was undertaken, as well as the number of PV^+^ synapses on to layer 5 neurons. The data from these assessments showed no difference between saline and IL-1β treatment groups for any metric (Fig. 4B–E), and therefore further analysis related to potential effects of montelukast was not performed.

Fibre orientation and myelination are strong drivers of FA and were therefore also assessed. There was no difference in total area of myelin staining between treatment groups (Additional file 1: Fig. S3G). However, directionality of axonal fibres extending into the cortex was significantly perturbed by IL-1β challenge in layers 5 and 6 (lower cortical layer) of the GM (*p* < 0.05), with a reduction of fibres in the radial direction. This was normalised with montelukast (Fig. 4F, G); layers 2–4 (upper cortex) were unaffected.

To determine whether the positive effects of montelukast in ameliorating elements of brain injury went beyond the cortex, an assessment of hippocampal GM and major WM tracts was performed. WM injury has been previously described during the postnatal period in this model [33]. This delayed myelination was not detected at P10 by immunohistochemistry, but gene expression for myelin proteins at this age did show a significant reduction in animals treated with IL-1β that was not ameliorated by high-dose montelukast treatment (Additional file 1: Fig. S3A–F). No long-term WM injury was detected with either immunohistochemistry or MRI in the cohort of animals assessed in this study (Additional file 1: Fig. S3G–I). Nor was any long-term hippocampus injury detected in these animals using either dMRI metrics (Additional file 1: Fig. S4A–D) or histological assessments (Additional file 1: Fig. S4E–O).

### Montelukast restores behaviour in mice with inflammation-induced brain injury

**Fig. 5:**
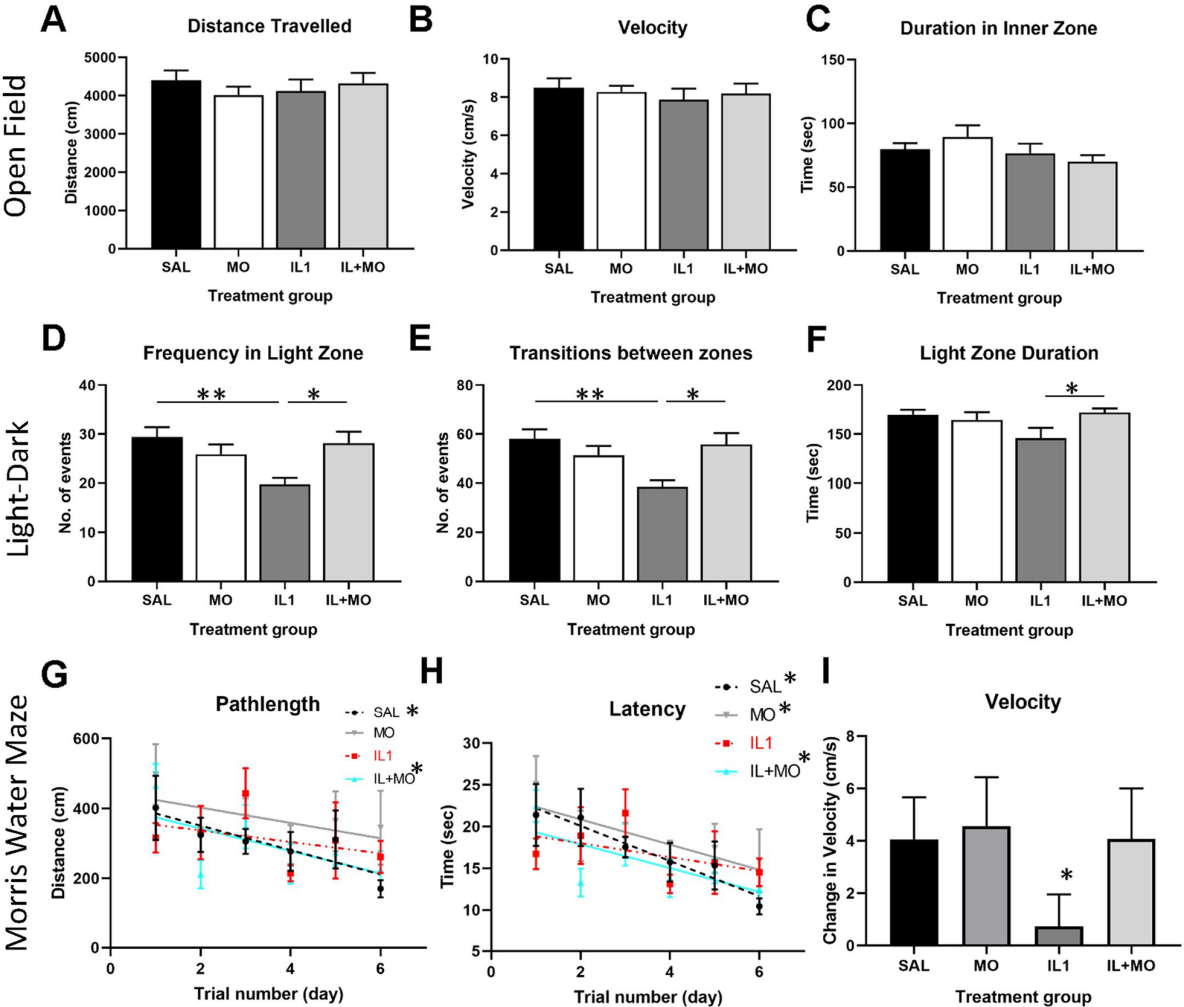
Montelukast ameliorates inflammation-induced behavioural abnormalities. A series of behaviour tests were performed to assess the capacity of montelukast to rescue behaviour deficits evident in this animal model and preterm infants. Open field data showed that the inflammatory model of preterm brain injury did not induce any motor deficits, with distance moved (**A**) and velocity (**B**) the outer zone, and time spent in the inner zone (**C**) comparable across all groups. The IL-1β treatment group exhibited anxiety-like behaviour in the light–dark box paradigm (**D**–**F**), which was resolved by treatment with montelukast. The Morris water maze was used to assess spatial learning; path length (**G**), latency (**H**) and velocity (**I**) to the submerged platform were assessed. IL-1β treatment reduced spatial learning as animals showed worsened rate of improvement in path length and latency to the platform with increasing trials. This learning deficit was ameliorated by montelukast treatment, with animals showing improved rates of learning. Data presented as mean ± SEM, *n* = 12 for all groups, *p < 0.05, **p < 0.01, ***p < 0.001. SAL: saline; IL1: IL-1β; IL + MO: IL-1β + montelukast; MO: montelukast

In this model of preterm brain injury, memory deficits have been shown that are comparable to those observed in human infants [33, 34]. Therefore, we investigated whether the low-dose montelukast was able to restore these, and other behaviours associated with neurodevelopmental disorders (Fig. 5). In the open field, there were no significant differences in the distance moved or velocity the outer region of the apparatus, across the 4 groups, confirming there are no significant locomotor deficits in this model (*p* > 0.05, Fig. 5A–C). There were no differences in the time spent in the inner zone of the open field between any of the groups.

IL-1β-treated animals exhibited anxiogenic behaviours in the light–dark box, indicating that this model of preterm brain injury can induce an anxiety phenotype (Fig. 5D–F); the frequency of the animals in the light zone (SAL: 29.4 ± 2.0, IL1: 19.8 ± 1.3), as well as the number of transitions between the light and dark zones (SAL: 58.0 ± 3.9, IL1: 38.5 ± 2.7), were significantly decreased. This behaviour deficit was ameliorated by montelukast (frequency: IL + MO: 28.2 ± 2.3, *p* = 0.005; SAL vs IL1 p = 0.004, IL1 vs IL + MO *p* = 0.014; transitions: IL + MO: 55.8 ± 4.7, *p* = 0.004; SAL vs IL1 *p* = 0.003, IL1 vs IL + MO *p* = 0.011). As with the open field test, there were no general changes in locomotion that could have confounded this finding (Additional file 1: Fig. S5A, B).

In the MWM, saline-treated animals showed significant decreases in path length (*p* = 0.018) and latency (*p* = 0.001) to platform, and increase in velocity (swim speed, *p* = 0.024) over the multiple trials and days of the experiment (linear regression analysis, Fig. 5G–I, Additional file 1: Fig. S5). By comparisons, the IL-1β group did not show significant spatial learning in the MWM (*p* = 0.31 for path length, *p* = 0.19 for latency, *p* = 0.54 for velocity). Animals treated with montelukast showed improved learning; with increasing trials, there were significant decreases in the distance travelled and latency to reach the platform (*p* = 0.017 and *p* = 0.004, respectively, linear regression analysis) at a rate comparable to the saline group (Fig. 5G, H; Additional file 1: Fig. S5C). Finally, the change in animal velocity over the course of the experiment was decreased with IL-1β challenge, which was normalised with montelukast (Fig. 5I, Additional file 1: Fig. S5D).

Overall, these data suggest that the inflammation model of preterm brain injury induces anxiety and spatial learning deficits that are ameliorated by montelukast at a dose of 3 mg/kg.

## Discussion

Preterm brain injury causes life-long cognitive, motor and behavioural deficits. Most studies have focused on WM injury, and, to date, no treatments have progressed to the clinic [38]. Recently, GM injury has been recognised as an important primary injury in EoP, which opens the scope for therapeutic development. Data presented in this study show improvement in GM deficits in a model of preterm brain injury following treatment with montelukast, a cysteinyl leukotriene receptor antagonist. Specifically, in this inflammatory model of mild perinatal brain injury, montelukast reduced the precipitating inflammatory event and attenuated the interneuronopathy. Furthermore, the increased MD detected in the adult cortex with injury was not detected in the animals that receive montelukast concomitant with the inflammatory injury. In turn, montelukast was able to normalise behavioural deficits, suggesting a functional link between GM deficits and some behavioural outcomes in preterm infants. We therefore propose montelukast as a potential therapy to ameliorate GM injury and associated behavioural deficits.

Montelukast was specifically chosen as a potential therapeutic agent for this condition as, in addition to being widely used and safe in children, it regulates a branch of the inflammatory response not constitutively active within the brain. Neuropoietic cytokines are important for providing a number of proliferation, differentiation and migration signals in the developing brain (reviewed by [32, 87]), which are fundamental for normal brain development. The CystLR1 and CystLR2 GPCR receptors are not constitutively expressed in the brain but are upregulated with injury [58, 59], allowing regulation of injury without affecting normal developmental processes. Of note, the G protein-coupled orphan receptor GPR17 has recently been identified as contributing to oligodendrocyte and myelination [48, 88], but understanding of its role in injury is limited.

Montelukast was successful at ameliorating the established central and, to a lesser degree, peripheral pro-inflammatory response. It is still unclear how inflammation is specifically signalled from the peripheral to the central compartment of the body in this perinatal injury model, and whether montelukast exerts its effects directly on the brain. There were small but significant increases in the expression of CysLTR1, the receptor target for montelukast, and CysLT concentrations in the brain following systemic inflammation that suggest montelukast could have its actions centrally, should it be able to access the receptors. Age and brain region-specific alterations in blood–brain barrier permeability have previously been reported in models of perinatal brain injury [50, 89, 90], though chronic systemic inflammation has previously been shown to induce central inflammation irrespective of blood–brain barrier function [91]. Here, we found no significant alteration in blood–brain barrier permeability, suggesting inflammatory mediators cross the intact barrier or generate central inflammation following secretion from the cerebrovasculature [92]. Montelukast may modulate this process through either peripheral or central actions. Montelukast was considered to be excluded from the brain (FDA, montelukast, Singulair®, application number 20-829), though a limited, but still significant entry to the brain has been reported that could explain its neuroprotective effects [93]. Consistent with this, in the present study, montelukast was detected in the brain, further supporting the possibility of a direct effect. However, the amount of montelukast detected was a small fraction of that in the plasma, and the upregulation of the CysLTR1 is also small, reducing the likelihood that the primary action is direct to the CNS. Based on these data, we hypothesis that the primary mechanisms for action of montelukast in this mouse model is to modulate the signalling of inflammation from the periphery to the brain, mostly likely at the blood–brain barrier. The greater effect of montelukast on the central, rather than peripheral, inflammatory response, and the relatively low production of leukotrienes and entry of montelukast to the brain support this hypothesis. Reducing the inflammation signalled to the brain will result in downstream therapeutic benefits in the CNS resulting from less central inflammation, and the reduction of short- and long-term neuropathology suggest a prevention or amelioration of some aspects of the injury, rather than a regenerative response. Further work is required to test this hypothesis, and to determine the clinical situations in which this preventative approach is appropriate. As the amelioration of the peripheral response was not complete, we also hypothesise that adjunct anti-inflammatory therapy could be advantageous in a future clinical treatment regimen. However, central inflammation, the key driver of injury in this model, was substantially reduced as early as 4 h after injury induction, which likely contributes to preventing the later decreases in parvalbumin interneuron number and behavioural deficits.

Injury to cortical parvalbumin interneurons has previously been described in this model [13], a cell type that has been shown to be selectively sensitive to inflammation [94]. Parvalbumin interneurons are the largest population of interneurons in the cortex and play an important role in integrating excitatory and inhibitory neuronal signalling. Altered parvalbumin function in the brain has been associated with a range of behavioural deficits, including memory dysfunction and impaired social interaction, repetitive behaviours, and increased seizure risk [95, 96]. Critically, the number of parvalbumin interneurons was normalised by montelukast as early as P10, indicating that early treatment can prevent GM injury caused by inflammation-associated preterm brain injury.

Similarly, an increased MD in the cortex in the inflammatory-injury group was not detected with montelukast treatment. Interestingly, a decrease in FA was observed only with a combination of inflammatory injury and montelukast treatment, compared to saline-treated controls. Assessments of normal early human [18, 97] and rodent cortical development [98] show that FA and MD decrease with increased cellular complexity, cell number and arborisation. Consistently, studies have shown increased MD as a result of cell loss [98, 99] or reduced arborisation [10]. In the present study, we have seen no evidence of extensive cell loss, and our previous studies using this model have not shown changes in cortical arborisation [13]. It is likely that more long-term neuropathological changes exist than have been identified in this study, as the differences observed with diffusion MRI metrics in the cortex between treatment groups are not easily explained by the histological findings of this study. Of note, MD can detect loss of a relatively small proportion of cells, and it has also been hypothesised that reduced size of myelinated axons can be detected with this metric [99]. This may explain both why long-term neuropathological correlates of this injury have not been detected to date and the slight (non-significant) decrease in FA in the cortex of the inflammatory-injury group. Petrenko et al. [99] also suggest some compensatory increase in arborisation of remaining cells after low level cell loss. It is, therefore, also possible that the FA result in our study reflects an undetected increase in dendritic arborisation driven by a combined action of inflammatory injury and montelukast treatment.

Montelukast was able to protect from inflammation-induced behavioural deficits in a mouse model of EoP. Improvements in anxiety and spatial learning were evident, which coexisted with the normalisation of parvalbumin interneurons early in development, and long-term organisation of cortical myelinated fibres as well as absence of the inflammation-induced increase in MD. Collectively, these findings suggest that GM injury may play a greater role in EoP than previously appreciated. Certainly, deficits in parvalbumin interneurons have been associated with these specific behaviour changes [94, 100]. In preterm infants, changes in cortical volume, cortical folding and microstructural connectivity have all been reported, which correlated with poor behavioural outcomes [15, 17, 101]. This suggests that subtle, early injury, such as the transient and selective parvalbumin interneuronopathy seen here, may be important factors in influencing outcome, and essential targets for therapy.

Work remains to determine the clinical situations in which this preventative approach is most appropriate, as some of the inflammatory drive may start prior to birth. Our hypothesis suggests that preventing inflammatory signalling from the periphery to the brain can limit down stream injury but does require therapeutic targeting of this inflammatory response early in the disease process. This can be tested in our preclinical mouse model and should be considered for meaningful evaluation of montelukast’s potential in future clinical trials. Of note, this preventative/ameliorative action was not seen on the early white matter injury, suggesting either an increased susceptibility of myelin to inflammatory injury that was not completely ameliorated or an interaction between montelukast and the developing oligodendrocytes that warrants further investigation. While this hypothesised mechanism is of scientific interest, and will be investigated, the key finding of ameliorated neuropathology is clearly evidence of the potential utility of this therapeutic approach. Future studies should also consider the broader beneficial potential of montelukast of other inflammation-associated pathologies in the preterm infant [102].

## Conclusions

In conclusion, this study has demonstrated the importance of GM injury in EoP and that montelukast is a safe, repurposable drug. Montelukast is an effective treatment at reducing central inflammation, normalising parvalbumin interneuron numbers, and behavioural deficits associated with EoP in an inflammatory model of preterm brain injury. We therefore propose that montelukast has potential as a novel therapy for preterm brain injury.

## Supplementary Information

**Additional file 1: Figure S1.** Effect of montelukast on CysLTR1 and CysLT in the brain. **Figure S2.** IL-1β induces vascular inflammation, but not BBB breakdown. **Figure S3.** No improvement of white matter damage is evident following montelukast treatment in this model. **Figure S4.** No hippocampal damage evident in this model. **Figure S5.** Inflammation and/or montelukast treatment does not induce motor deficits.

## Data Availability

The data that support the findings of this study are available from the corresponding author, HBS, upon reasonable request.

## Abbreviations

ABP: Avidin–biotin–peroxidase
AC: Anterior commissure
AD: Axial diffusivity
ANOVA: Analysis of variance
CA: Cornu ammonis
CC: Corpus callosum
CNPase: 2’,3’-Cyclic-nucleotide 3’-phosphodiesterase
CysLTs: Cysteinyl leukotrienes
CysLTR: Cysteinyl leukotriene receptor
d: Diffusion
DAB: 3,3′-Diaminobenzidine
DG: Dentate gyrus
DTI: Diffusion tensor imaging
EoP: Encephalopathy of prematurity
EC: External capsule
EPI: Echo planar imaging
FA: Fractional anisotropy
FSL: FMRIB Software Library
GAPDH: Glyceraldehyde 3-phosphate dehydrogenase
GFAP: Glial fibrillary acidic protein
GM: Grey matter
H&E: Haematoxylin and eosin
HRP: Horseradish peroxidase
ICAM1: Intracellular adhesion molecule 1
IHC: Immunohistochemistry
IL: Interleukin
i.p.: Intraperitoneal
MBP: Myelin basic protein
MD: Mean diffusivity
MO: Montelukast
MRI: Magnetic resonance imaging
MWM: Morris water maze
NDI: Neurite density index
NHS: Normal horse serum
ODI: Orientation dispersion index
P: Postnatal
PBST: Phosphate buffered saline + Tween 20
PFA: Paraformaldehyde
PMA: Post-menstrual age
PV: Parvalbumin
qRT-PCR: Quantitative real time polymerase chain reaction
ROI: Region of interest
SAL: Saline
SEM: Standard error of the mean
TE: Echo time
TNF: Tumour necrosis factor
TR: Repetition time
WM: White matter
