# Supplementary material for: Montelukast reduces grey matter abnormalities and functional deficits in a mouse model of inflammation-induced encephalopathy of prematurity
Source: J Neuroinflammation. 2022 Oct 29;19:265. doi: 10.1186/s12974-022-02625-5 (PMC9617353; doi:10.1186/s12974-022-02625-5)
Supplement: Supplementary file 1 — Additional file 1: Figure S1. Effect of montelukast on CysLTR1 and CysLT in the brain. Figure S2. IL-1β induces vascular inflammation, but not BBB breakdown. Figure S3. No improvement of white matter damage is evident following montelukast treatment in this model. Figure S4. No hippocampal damage evident in this model. Figure S5. Inflammation and/or montelukast treatment does not induce motor deficits. [file 12974_2022_2625_MOESM1_ESM.pdf]

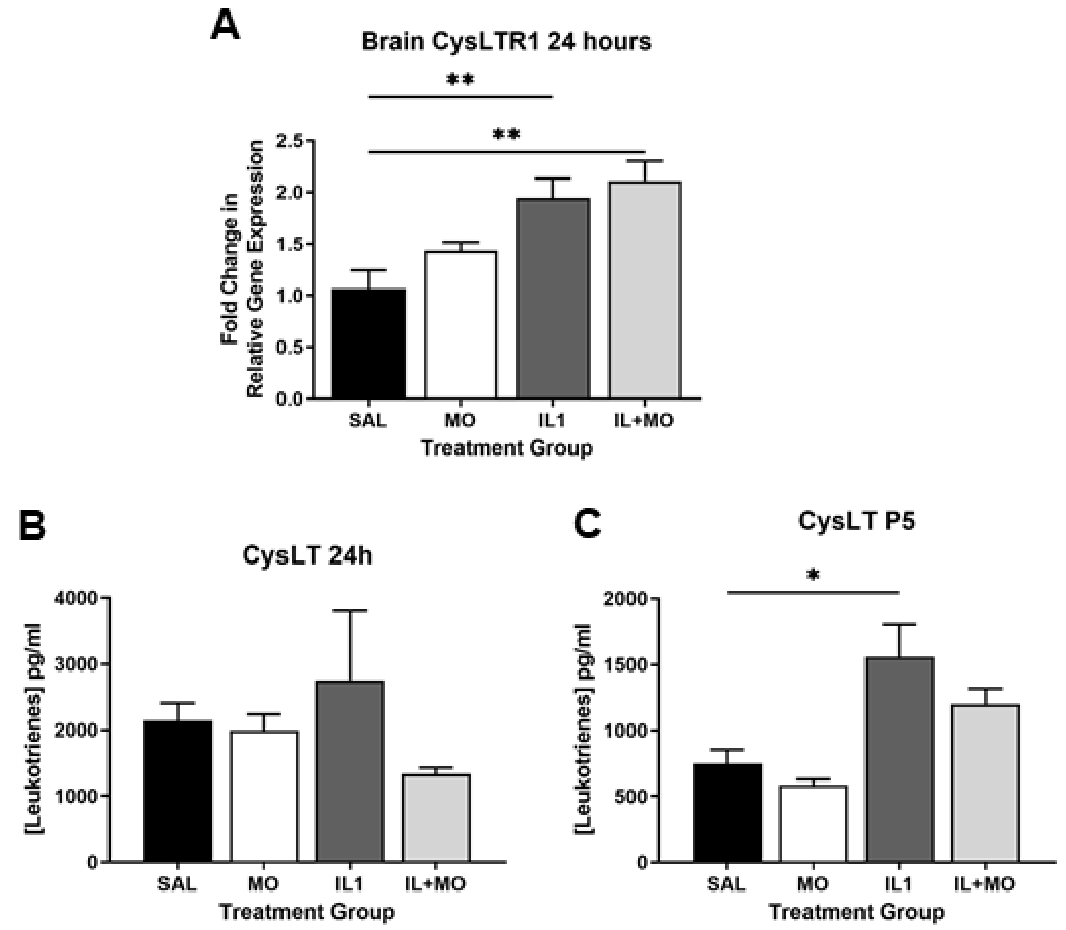

Figure S1. Effect of Montelukast on CysLTR1 and CysLT in the brain. Relative quantification of CysLTR1 transcript expression within brains of mice 24 hours after treatment (A). Significantly higher expression was detected in both groups treated with IL-1β, irrespective of Montelukast treatment. ELISA quantification of leukotriene proteins extracted from the brain 24h or a P5 following treatment (B, C). IL-1β treatment resulted in significantly increased levels of proteins at P5. Results are expressed as mean ± SEM. Asterisks indicate a significant difference between two treatment groups, *$p<0.05$ and **$p<0.01$. SAL – Saline (n=6 gene expression, CysLT quantification: n=4 at 24h, n=5 at P5), IL1 – IL-1β (n=7 gene expression, CysLT quantification: n=5 at 24h and P5), IL+MO – IL-1β + Montelukast (n=8 gene expression, CysLT quantification: n=6 at 24h, n=5 at P5), MO – Montelukast (n=8 gene expression, CysLT quantification: n=3 at 24h, n=2 at P5). CysLT – Cysteinyl Leukotrienes, CysLTR1 – Cysteinyl Leukotriene Receptor 1.

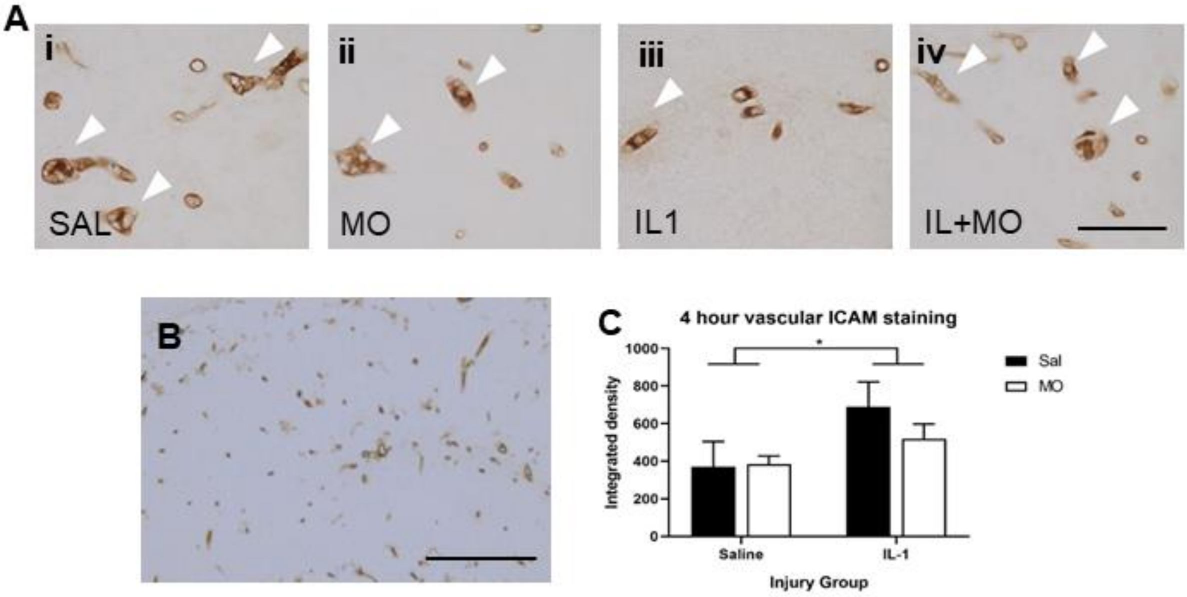

Figure S2. IL-1β induces vascular inflammation, but not BBB breakdown. Immunostaining for adhesion molecule ICAM or serum albumin was used to evaluate vascular inflammation and BBB breakdown, respectively. Serum staining did not reveal any evidence of BBB breakdown in this model of preterm brain injury (A). IL-1β-treated animals had increased ICAM staining (B, C), which was not significantly ameliorated by Montelukast. Data presented as mean ± SEM, SAL – Saline (n-6), IL1 – IL-1β (n-6), IL+MO – IL-1β + Montelukast (n-7), MO – Montelukast (n-6).

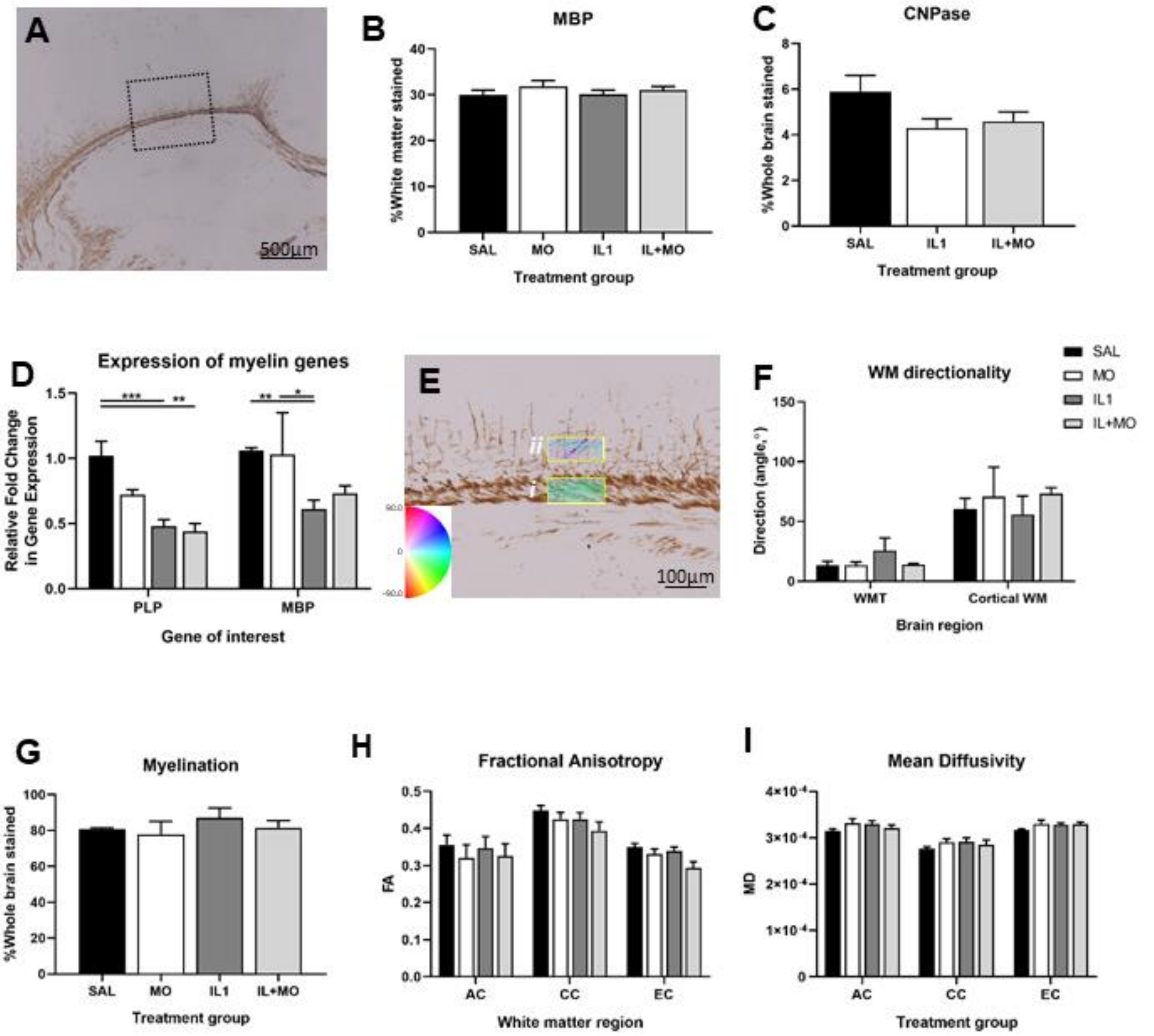

Figure S3. No improvement of white matter damage is evident following Montelukast treatment in this model. White matter area was assessed from immunohistochemcal staining of the brain (A) and quantified for myelin basic protein (MBP, B) and CNPase (C), showing no significant difference with inflammation or Montelukast treatment. Expression of myelin genes was determined with qRT-PCR (D), and showed significant reductions with inflammatory injury, but no amelioration with Montelukast treatment (D). Directionality analysis of external capsule and white matter tracts protruding into the cortex (E-F) did not show a significant difference between groups. dMRI of the adult brain and quantification of DTI metrics within major white matter tracts (anterior commissar, AC; corpus callosum, CC; external capsule, EC) showed no long pathology, consistent with previous findings in this

model. Data presented as mean ± SEM, *p<0.05. Scale bars E = 100µm. SAL – Saline (P10: n=5 histology, n=4 qRT-PCR; P40: n=7 MRI, n=3 histology), IL1 – IL-1β (P10: n=8 histology, n=7 qRT-PCR; P40: n=9 MRI, N=3 histology), IL+MO – IL-1β + Montelukast (P10: n=6 histology, n=3 qRT-PCR; P40: n=8 MRI, n=4 histology), MO – Montelukast (P10: n=5 histology, n=2 qRT-PCR; P40: n=6 MRI, n=3 histology).

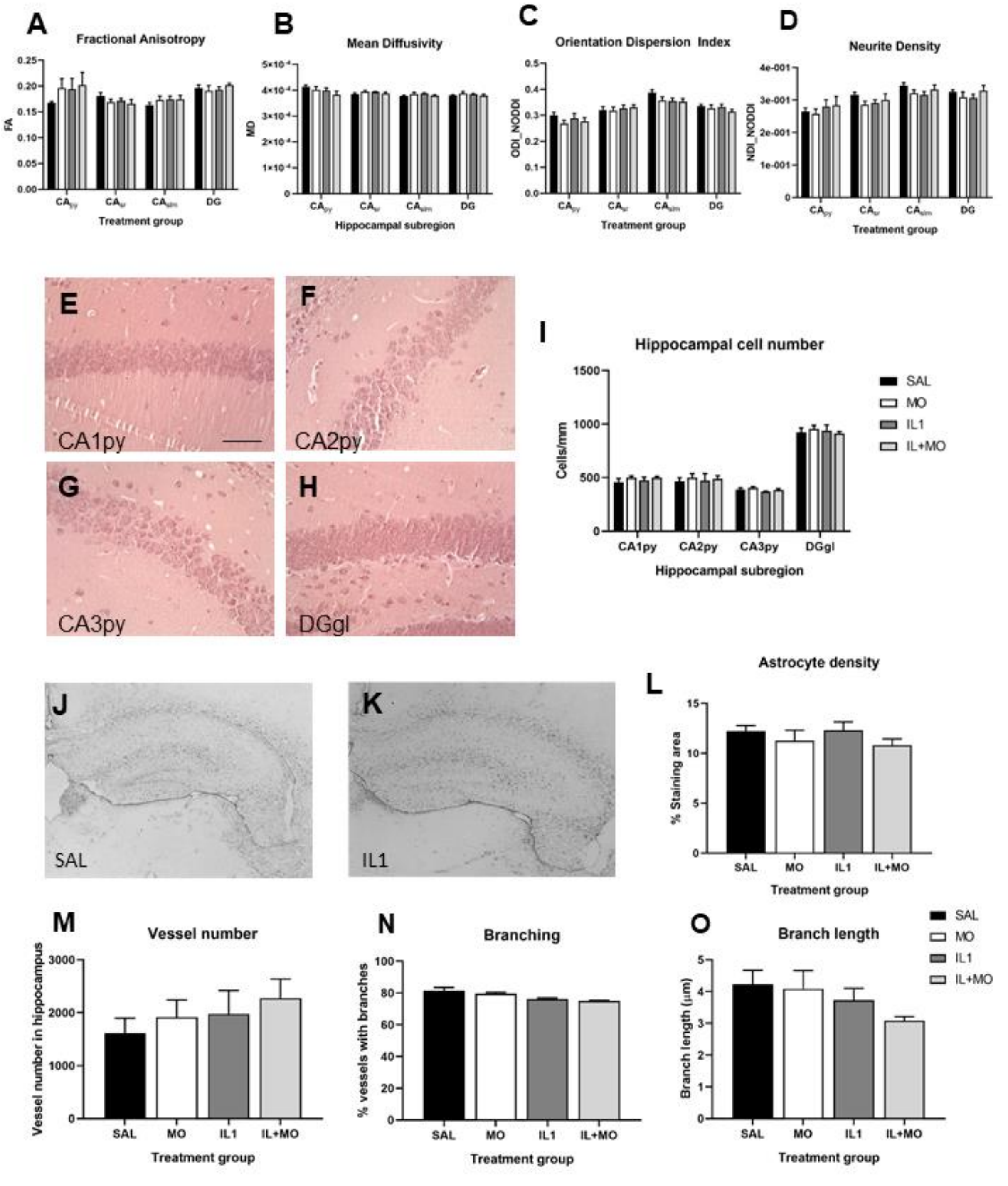

Figure S4. No hippocampal damage evident in this model. MRI and histological analysis of the hippocampus was completed at P40 to identify any injury that may contribute with cortical pathology to explain behavioural deficits. MRI analysis showed no change in FA (A), MD (B), ODI (C), or NDI (D) for any hippocampal layer (CA_{py}, pyramidal layer; CA_{sr}, stratum radiatum; CA_{slm}, stratum lacunosum moleculare; DG, dentate gyrus). H&E staining revealed

no difference in cell density across the 4 groups (A-I). GFAP staining of astrocytes similarly showed no changes (J-L). Blood vessel number (M), branching (N) and length (O) were also unaffected. Data presented as mean ± SEM. SAL – Saline (n=7 MRI, n=3 histology), IL1 – IL-1β (n=9 MRI, N=3 histology), IL+MO – IL-1β + Montelukast (n=8 MRI, n=4 histology), MO – Montelukast (n=6 MRI, n=3 histology).

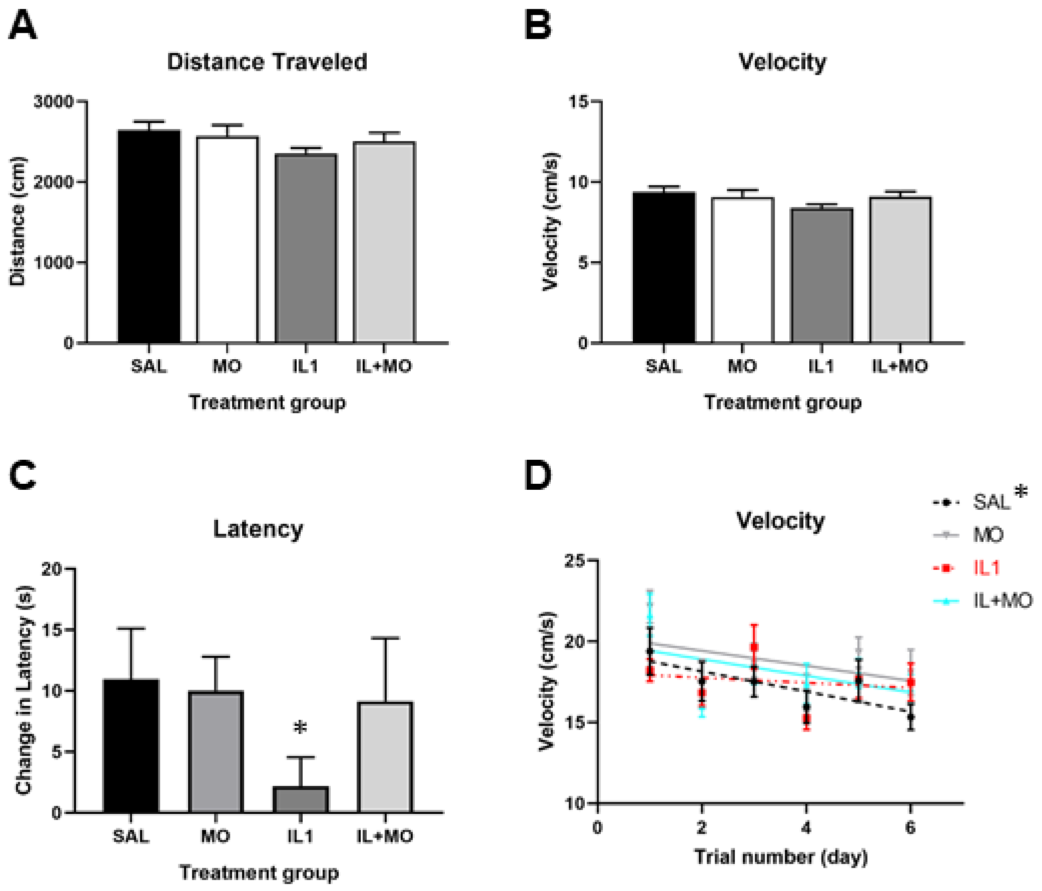

Figure S5. Inflammation and/or Montelukast treatment does not induce motor deficits. The IL-1β treatment group exhibited anxiety-like behaviour in the light-dark box paradigm, but no disruption in normal motor behaviours such as distance travelled (A) and velocity (B). Morris water maze average escape latency (C) and velocity to find platform data (D). presented as time per trial over the 6-day spatial memory test. Data presented as mean ± SEM, n=12 for all groups. SAL – Saline, IL1 – IL-1β, IL+MO – IL-1β + Montelukast, MO – Montelukast.
